# Evaluation of Lymphatic and Vascular Invasion in Relation to Clinicopathological Factors and Treatment Outcome in Oral Cavity Squamous Cell Carcinoma

**DOI:** 10.1097/MD.0000000000001510

**Published:** 2015-10-30

**Authors:** Mohamad Adel, Huang-Kai Kao, Cheng-Lung Hsu, Jung-Ju Huang, Li-Yu Lee, Yenlin Huang, Timothy Browne, Ngan-Ming Tsang, Yu-Liang Chang, Kai-Ping Chang

**Affiliations:** From the Department of Otorhinolaryngology–Head and Neck Surgery (MA,K-PC); Department of Plastic and Reconstructive Surgery (H-KK,J-JH,TB); Division of Hematology-Oncology, Department of Internal Medicine (C-LH); Department of Pathology (L-YL,YH); Department of Radiation Oncology (N-MT); Department of Oral and Maxillofacial Surgery, Chang Gung Memorial Hospital (YLC); School of Medicine, College of Medicine, Chang Gung University, Tao-Yuan, Taiwan (C-LH,N-MT,K-PC); and Division of Surgical Oncology, Al-Azhar Faculty of Medicine, Al-Azhar University Hospitals, Cairo, Egypt (MA).

## Abstract

This study evaluated the associations between lymphatic and vascular invasion of oral cavity squamous cell carcinoma (OSCC) and clinicopathological manifestations, as well as their impact on patient outcomes after treatment.

In total, 571 patients with primary OSCC who underwent surgery with or without adjuvant therapy were enrolled.

Lymphatic and vascular invasion were found in 28 (5%) and 16 (3%) patients, respectively. Significant associations were found between lymphatic and vascular invasion and overall stage (*P* < 0.001 and *P* = 0.020, respectively), tumor stage (*P* = 0.009 and *P* = 0.025, respectively), nodal metastasis (both *P* < 0.001), extracapsular spread (both *P* < 0.001), perineural invasion (both *P* < 0.001), bone invasion (*P* = 0.004 and *P* = 0.001, respectively), depth of invasion (*P* < 0.001 and *P* = 0.001, respectively), and pathologic differentiation (*P* = 0.002 and *P* < 0.001, respectively). In the analysis of adverse events during follow-up, neither lymphatic nor vascular invasion was statistically associated with local recurrence, neck recurrence, and distant metastasis. Although lymphatic invasion exhibited significant associations with poorer overall survival (*P* < 0.001), disease-specific survival (*P* < 0.001), and disease-free survival (*P* = 0.01), it was not demonstrated to be an independent prognostic factor in all multivariate analyses.

Although both lymphatic and vascular invasion are associated with many clinicopathological manifestations, neither affects the occurrence of locoregional recurrence and distant metastasis in patients with OSCC after treatment.

## INTRODUCTION

Oral cavity squamous cell carcinoma (OSCC) is the most common oral malignancy, representing 90% of all malignant neoplasms of the oral cavity.^1^ OSCC is a multifactorial disease, and among the most prominent determinant factors for disease progression and prognosis are the histopathological features of the primary site of the tumor. Numerous studies have demonstrated that tumor size, histological differentiation, nodal stage, and perineural invasion can predict recurrence and survival in patients with OSCC.^2–5^ However, representing an important step in tumor progression toward regional and distant metastasis, the impact of lymphatic and vascular invasion on prognosis and survival has not yet been fully clarified. Although they have long been considered important histopathological features for locoregional recurrence or even distant metastasis, currently available cohort studies have not fully addressed the role of lymphatic and vascular invasion in terms of clinicopathological factors and treatment outcomes.

To the best of our knowledge, no cohort study examined lymphatic and vascular invasion separately in terms of the prognosis and survival of patients with OSCC. The aim of this cohort study was to examine the associations of lymphatic and vascular invasion with other clinicopathological manifestations, such as perineural invasion and extracapsular spread, the failure patterns after OSCC treatment, and the outcome of patients with OSCC who were treated by surgical resection as the primary modality with or without the use of adjuvant treatment. The current study also evaluated whether lymphatic and vascular invasion may provide supplemental prognostic value in addition to the current TNM system. In addition, the prognostic value of lymphatic and vascular invasion for predicting locoregional control, distant metastasis, and overall survival was also evaluated.

## METHODS

### Patient Characteristics

Between August 2002 and December 2012, 571 consecutive patients who were diagnosed with OSCC and who underwent surgery as the primary modality of therapy at Chang Gung Memorial Hospital (Linko Medical Center, Tao-Yuan, Taiwan) were enrolled in the study. All patients were followed up for at least 18 months or until death. Patients with at least one of the following conditions were excluded from the study: any prior history of malignancy, unresectable disease, other primary cancer (synchronous or metachronous), recurrent cancer, distant metastasis at presentation, initial treatment with neoadjuvant therapy, or a medical contraindication for surgery. Lesions diagnosed as carcinoma in situ, verrucous carcinoma, or a histologically basaloid subtype were also excluded from the study. This study was approved by the hospital Institutional Review Board.

Patients in the study underwent standard preoperative workups according to institutional guidelines, including a detailed history, complete physical examination, computed tomography or magnetic resonance imaging, chest radiographs, bone scan, and abdominal ultrasonography. The primary tumors were excised with adequate surgical margins, and the tumor margin tissues were sent for cryosectioning and examination intraoperatively to ensure that the surgical margins were tumor free. Various types of neck dissection were performed according to the primary tumor site and clinical lymph node status.^6^ After surgical treatment, the pathological tumor and nodal staging (TNM) staging was performed according to the AJCC Cancer Staging 2010 guidelines to determine whether adjuvant treatment was indicated.^7^

Postoperative radiotherapy was performed on patients with pathologic T4 tumors and positive lymph nodes within 6 weeks following surgery. Patients with any pathologic findings such as metastasis in multiple neck lymph nodes, extracapsular spread, positive surgical margins, nodal dissemination at level 4 or 5, and perineural invasion received adjuvant concurrent chemoradiotherapy. The chemotherapy was a cisplatin-based regimen, and the total radiation dose was 66 Gy. The prescribed dose was delivered in fractions of 1.8–2 Gy per day for 5 days per week. All patients completed regular follow-up visits every 2 months for the first year after discharge, every 3 months for the second year, and every 6 months thereafter.

### Histopathology

**FIGURE 1:**
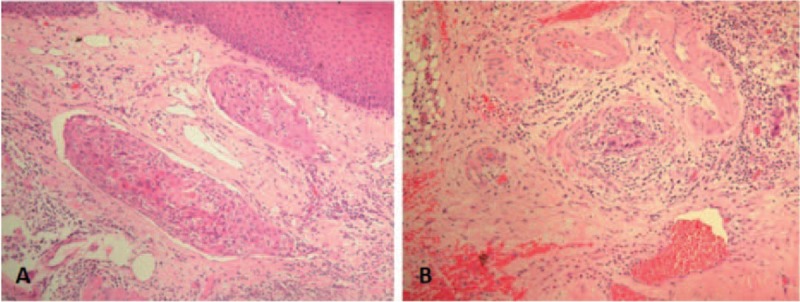
Histopathology (100×, H & E staining). A, Histological examination showed 2 foci of submucosal lymphatic permeation. B, Histological examination showed vascular invasion.

The histological differentiations were determined on the basis of the original pathology reports examined by multiple pathologists at this single institute. Two pathologists (L-YL and YH) were invited to review the specimens again independently for quality control of these pathological diagnoses. For each patient, we recorded the following data: anatomic subsite of the oral cavity, histopathological findings of tumor differentiation, bone invasion, perineural invasion, tumor depth, tumor status, lymph node status, overall stage, extracapsular spread status, lymphatic invasion, and vascular invasion. Lymphatic and vascular invasion were reported in the whole series based solely on hematoxylin and eosin staining (Figure 1). Lymphatic and vascular invasion were defined as the presence of aggregates of tumor cells within endothelial lined spaces with no underlying muscular walls and invasion of the media of a vessel with ulceration of the intima, respectively.^8–11^

### Data and Statistical Analysis

Patient and OSCC tumor characteristics were stratified according to various clinicopathological factors and evaluated using the *χ*^2^ test, Fisher exact test, Wilcoxon test, or the Kruskal–Wallis test, where appropriate. The factors evaluated included sex, age, TNM staging, and other pathological findings. The time to events, including local recurrence, neck recurrence, distant metastasis, and death, from the date of surgical removal of the primary tumor was calculated for each patient. Multivariate regression analyses were applied to define specific risk factors for overall survival (OS), disease-specific survival (DSS), and disease-free survival (DFS). Concerning the patients’ survival, the survival rate is estimated by Kaplan–Meier plotting and compared by log-rank test. For Kaplan–Meier plotting, in OS, the event is defined as the patient death directly from OSCC or from an unrelated cause. In DSS, the event is specified only as the death related to OSCC. In DFS, the event is defined as the patient death or any tumor relapse occurring loco-regionally or distantly. Statistical analyses were performed using SAS software (version 9.1; SAS Institute, Cary, NC). All patients received follow-up consultations at our outpatient clinic until July 2014 or death. All *P* values were 2-sided with the significance level set at *P* < 0.05.

## RESULTS

The age at diagnosis for the 571 patients with OSCC ranged from 21.9 to 86.8 years (median, 51.2 years). The study population consisted of 516 men and 55 women. The tumor subsites were the buccal mucosa (209 patients), gum (83), hard palate (15), lip (18), floor of the mouth (35), and tongue (211). Lymphatic invasion was reported in 28 (4.9%) patients, and vascular invasion was reported in 16 (2.8%) patients. Lymphatic invasion was not found to be associated with a particular sex (*P* = 0.842) or age group (*P* = 0.894). The same finding was noted for vascular invasion regarding sex (*P* = 0.693) and age (*P* = 0.218). The patient characteristics that correlated with the lymphatic and vascular invasion of OSCC are listed in Table 1.

**TABLE 1:**
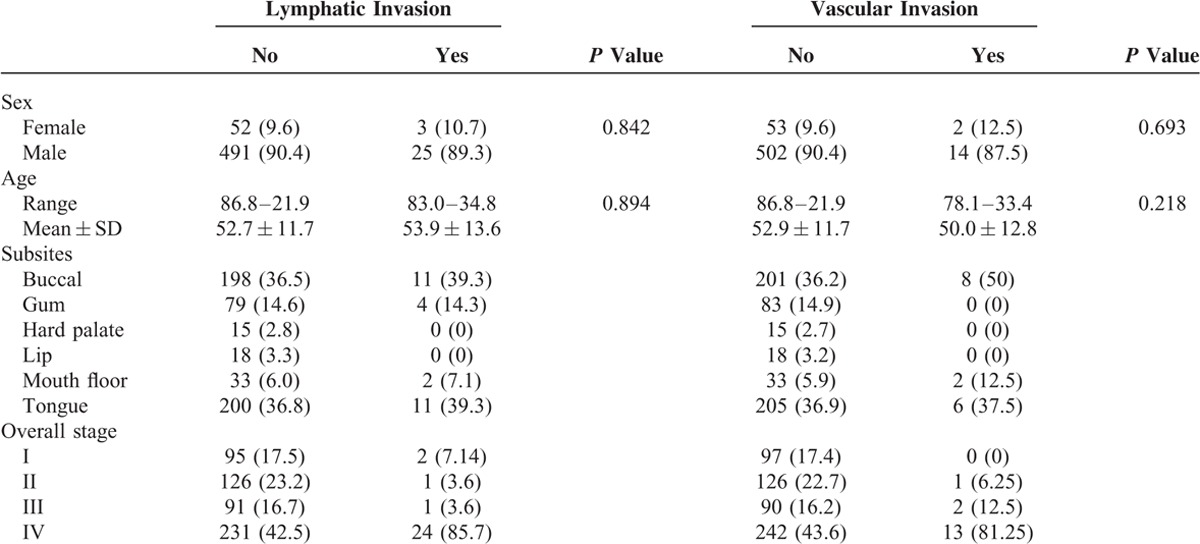
Patient Characteristics Correlated With the Lymphatic and Vascular Invasion of Oral Squamous Cell Carcinoma

Table 2 presents the association of lymphatic and vascular invasion with other clinicopathologic characteristics. There were significant associations between lymphatic invasion and T classification of the tumor (*P* = 0.009), nodal metastasis (*P* < 0.001), extracapsular spread (*P* < 0.001), perineural invasion (*P* < 0.001), bone invasion (*P* = 0.004), depth of invasion (*P* < 0.001), and pathologic differentiation (*P* = 0.002). Regarding vascular invasion, significant associations were noted with T classification (*P* = 0.025), nodal metastasis (*P* < 0.001), extracapsular spread (*P* < 0.001), perineural invasion (*P* < 0.001), depth of invasion (*P* = 0.001), and pathologic differentiation (*P* < 0.001), whereas no significant association was observed with bone invasion (*P* = 0.327). Overall, these associations indicated that the histopathological findings of lymphatic and vascular invasion in the primary OSCC tumors were associated with positivity for cervical metastasis, extracapsular spread, perineural invasion, poor differentiation, and deeper tumor depth.

**TABLE 2:**
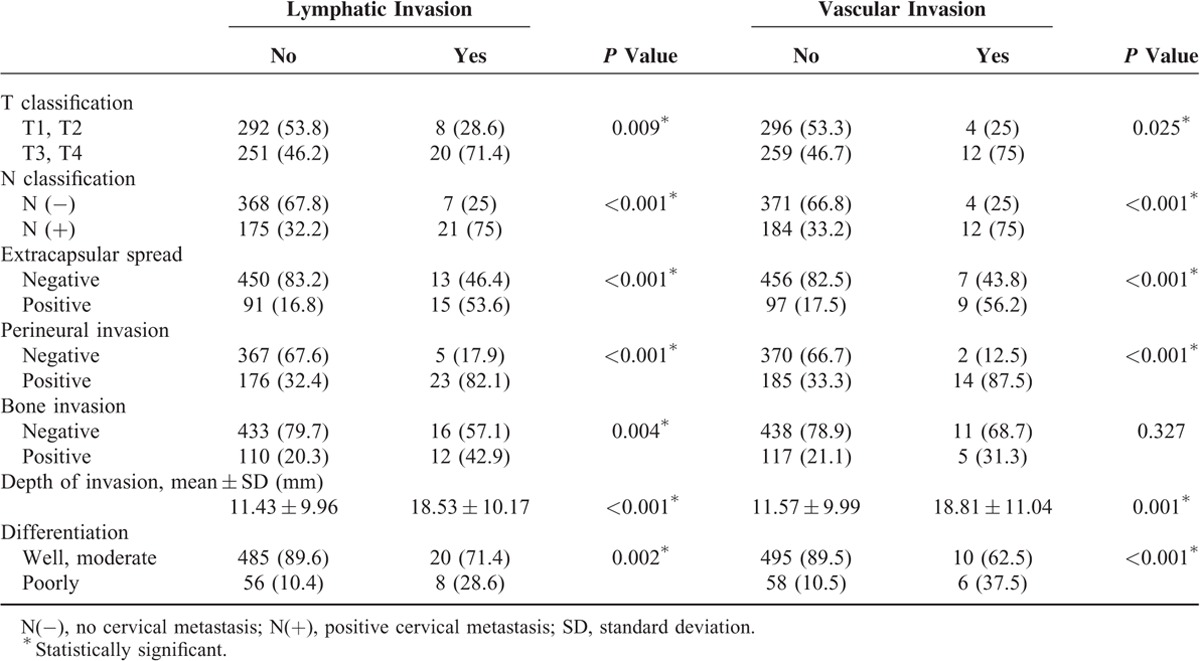
Clinicopathologic Characteristics of the Lymphatic and Vascular Invasion

In the analysis of adverse events affecting survival during follow-up, including local recurrence, neck recurrence, and distant metastasis, neither lymphatic nor vascular invasion was found to correlate with any of these variables. In addition, there was no difference in the duration from treatment to local recurrence, neck recurrence, and distant metastasis in the same comparison between the 2 groups (Table 3).

**TABLE 3:**
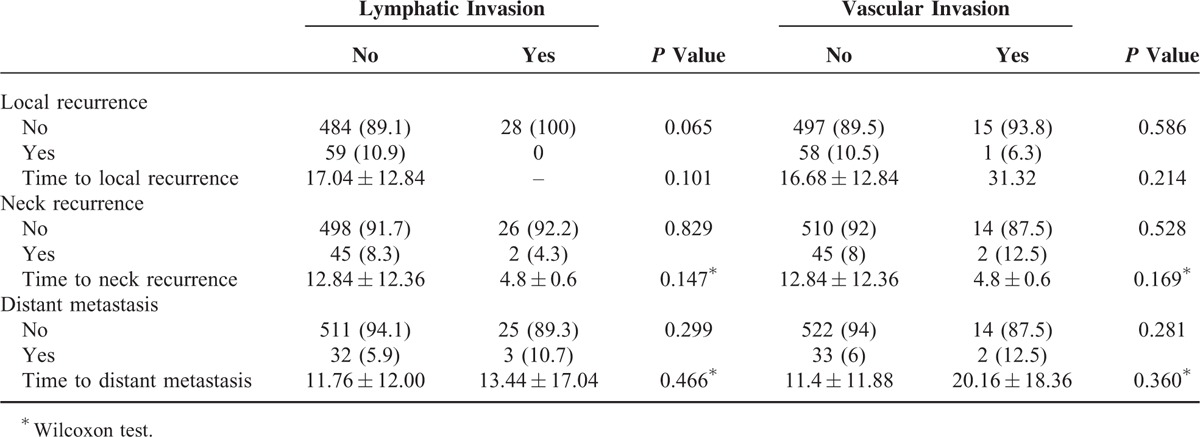
Correlation Between Lymphatic and Vascular Invasion and Adverse Events Impacting Survival During Follow-Up of OSCC

**FIGURE 2:**
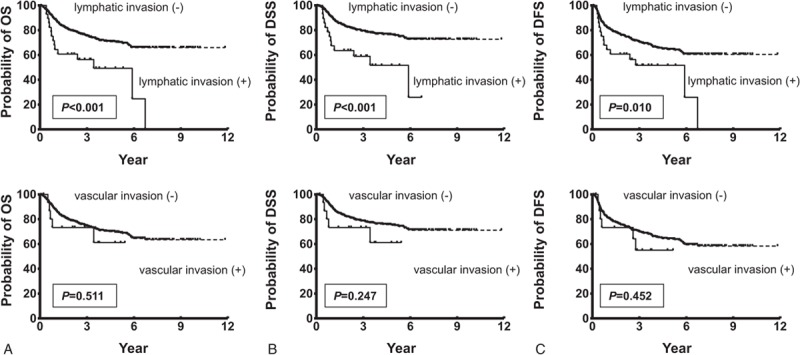
Association of lymphatic invasion with poorer patient survival. A, Kaplan–Meier plot for overall survival indicated that the 5-year overall survival (OS) rates for patients with and without lymphatic invasion were 49.3 and 70.3% (*P* < 0.001), respectively, but the difference in OS according to the presence of vascular was not significantly different between the groups (*P* = 0.511). B, The Kaplan–Meier plot for disease-specific survival (DSS) indicated that the 5-year DSS rates for patient subgroups were 51.6 and 76%, respectively (*P* < 0.001), but the difference in DSS according to the presence of vascular invasion was not significantly different between the groups (*P* = 0.247). C, The 5-year disease-free survival (DFS) rates for patients with and without lymphatic invasion were 51.6 and 64.4%, respectively (*P* = 0.010), but the difference in DFS according to the presence of vascular invasion was not significant between the groups (*P* = 0.452).

The association of lymphatic and vascular invasion with patients’ survival, including OS, DSS, and DFS, was further evaluated. Survival analysis revealed that the 5-year OS rates for patients with and without lymphatic invasion were 49.3% and 70.3%, respectively. These differences in OS were significant as compared by the log-rank test (*P* < 0.001; Figure 2A). However, patient OS according to the presence of vascular invasion was not significantly different between the groups (*P* = 0.511; Figure 2A). In DSS analyses, the Kaplan–Meier plots disclosed 5-year DSS rates for patients with and without lymphatic invasion of 51.6% and 76%, respectively. These differences were statistically significant, as observed by the log-rank test (*P* < 0.001; Figure 2B). On the contrary, the difference in DSS according to the presence of vascular invasion was not significantly different between the groups (*P* = 0.247; Figure 2B). Moreover, the 5-year DFS rates for patients with and without lymphatic invasion were 51.6% and 64.4%, respectively (*P* = 0.01; Figure 2C). Conversely, the difference in DFS according to the presence of vascular invasion was also not significantly different between the groups (*P* = 0.452; Figure 2C). To further ascertain whether lymphatic invasion can be applied as an independent predictor of patient survival, multivariate analysis was performed using age, sex, pT status, pN status, extracapsular spread, perineural invasion, tumor differentiation, tumor depth, lymphatic invasion, and vascular invasion as parameters in the Cox proportional regression model. Our results indicated that only pN status, extracapsular spread, and tumor stage are independent predictors of OS (*P* = 0.002, 0.010, and 0.048, respectively; Table 4), but lymphatic invasion (*P* = 0.133) and vascular invasion (*P* = 0.730) were not predictive of OS. Similar analyses also revealed that lymphatic invasion is also not an independent predictor of DSS and DFS (*P* = 0.920 and 0.750, respectively, data not shown).

**TABLE 4:**
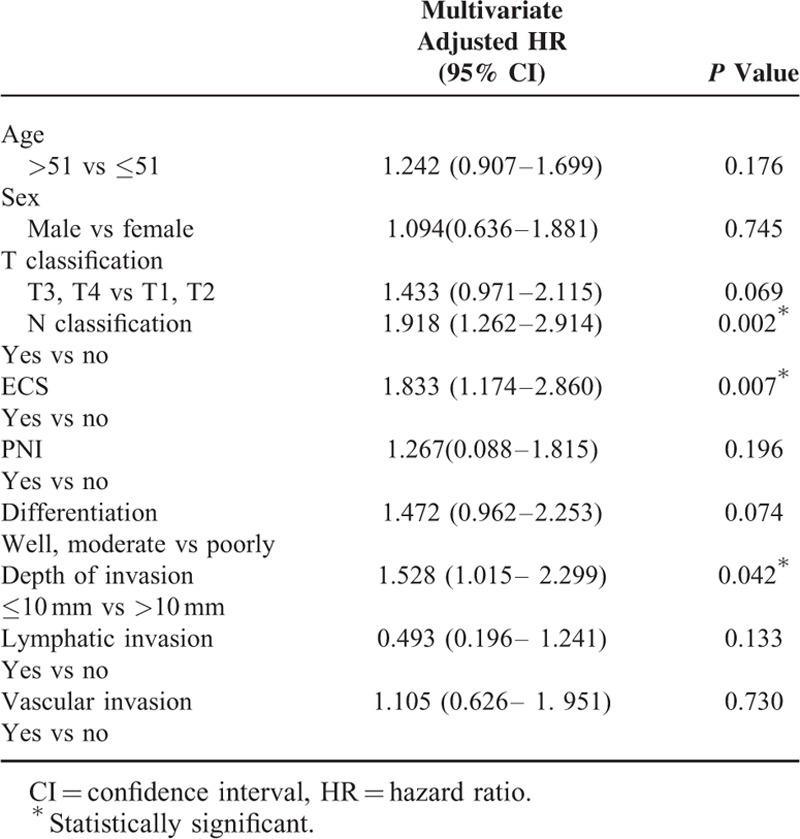
Cox Proportional Hazard Models for Overall Survival

## DISCUSSION

Tumor invasion into lymphatic and/or blood vessels has long been postulated to be an important pathologic factor. However, its impact on tumor locoregional control and recurrence remains to be elucidated. An assessment of the presence and extent of lymphovascular invasion was proposed by Jakobbson et al as a part of the multifactorial grading system.^12^ Its existence means that a considerable number of tumor cells are entering the vascular compartment, which is in turn one of the first steps for the potential development of metastasis.^13^ Jones et al reported the incidence of lymphovascular invasion in 69 patients with oral cavity carcinoma to be 35%, whereas Rahima et al reported an incidence of 15% in 101 patients with OSCC and oropharyngeal squamous cell carcinoma (SCC).^14,15^ Other investigators examined lymphovascular invasion in T1-T2 OSCC and reported an incidence in the range of 8% to 28%.^16,17^ The incidence of overall lymphovascular invasion in the current study was compatible with that in previous reports as lymphatic invasion was found in 28 (5%) patients, whereas vascular invasion was found in 16 (3%) patients. To the best of our knowledge, the current study contains the largest cohort of patients with OSCC reported to date in an attempt to analyze lymphatic and vascular invasion as 2 separate entities for examining their relationship with other clinicopathological in OSCC tumors and their impact on the survival and treatment outcome of the patients.

Poleksic et al first linked vascular invasion to aggressive tumor characteristics in head and neck SCC.^18^ Other studies reported the association of lymphovascular invasion with tumor site, thickness, perineural invasion, and status of resection margin.^8,9,19,20^ Additionally, many previous studies also found an association between lymphovascular invasion and cervical nodal metastasis,^9,20–25^ but a recent series did not find such association in T1-T2 cases of OSCC patients.^26^ The current study represents the first cohort study examining lymphatic and vascular invasion, respectively, in relation to clinicopathological characteristics. Lymphatic and vascular invasion were both significantly associated with T classification, N classification, perineural invasion, extracapsular spread, depth of invasion, and degree of differentiation.

The relation of lymphovascular invasion with the treatment outcome of OSCC remains unclear. Regarding the effect of lymphovascular invasion on treatment failure, Fagan et al reported that vascular and lymphatic invasion were not significantly associated with local recurrence in 142 patients with head and neck SCC.^27^ This finding was also supported by the observation of Chen et al in disease control of early-stage OSCC and of Tai et al in locoregional control of early-stage tongue SCC.^16,28^ However, other investigators have linked lymphovascular invasion with locoregional recurrence or distant metastasis.^17,29–31^ In the current study, the first examining the lymphatic and vascular invasion independently, we found that neither lymphatic nor vascular invasion impacts locoregional recurrence or distant metastasis after treatment. Furthermore, although some previous reports demonstrated an association of lymphovascular invasion with poorer patient survival,^1,8,14,19,32^ other reports showed no such association.^16,28^ In our study, vascular invasion was not found to affect patient survival, whereas lymphatic invasion was associated with worse OS, DSS, and DFS. In further evaluation of the prognostic significance by multivariate survival analysis, lymphatic invasion was not identified as an independent factor. These results suggest that lymphatic invasion is not an independent determinant of patient survival after controlling for all clinicopathological factors, whereas the depth of primary tumor invasion, nodal stage, and extracapsular spread are.

Efforts have been centered upon modalities for the postoperative management of patients with OSCC in light of their outcomes on an individual basis. Postoperative adjuvant radiotherapy is offered for patients with head and neck malignancies with large primary tumors or nodal metastasis. With evidence emerging from randomized control trials on the use of postoperative chemoradiotherapy in head and neck cancers, most centers administer postoperative chemoradiotherapy for patients with head and neck cancers and high-risk factors.^33^ Indeed, offering adjuvant radiotherapy to patients with low-stage OSCC tumors and concomitant risk factors would potentially expose a subpopulation to unnecessary, harmful toxicities, and this overtreatment would have negative effects on patient quality of life resulting from late adverse sequelae, such as neck soft tissue fibrosis, dysphagia, xerostomia, radionecrosis, or radiation-induced malignancies.^33,34^ Therefore, the precise identification of low-stage patients who are at high risk of disease progression or recurrence would represent a tremendous advantage in the management of this disease.^35^ Besides, according to the current study, lymphatic and vascular invasion both have no influence on the adverse events affecting locoregional control or distant metastasis during follow-up. Moreover, lymphatic invasion was not an independent prognostic factor in survival analysis. A large, prospective cohort study will be necessary to determine the efficacy of using vascular or lymphatic invasion as criteria for adjuvant chemotherapy and/or radiotherapy.

Because lymphatic and vascular invasion were discovered to be strongly associated with various pathological factors mentioned above (Table 2), we found that most OSCC patients with lymphatic or vascular invasion received postoperative adjuvant radiotherapy or chemoradiotherapy under our preexisting protocol. Twenty-six (93%) of 28 and 13 (81%) of 16 OSCC patients with lymphatic and vascular invasion received postoperative adjuvant treatments, respectively. In other words, only 2 cases of OSCC patients with lymphatic invasion and 3 cases with vascular invasion did not receive any postoperative treatment. Due to these limited numbers, we could not analyze the survival benefit of postoperative treatment in the patients with these 2 pathological factors.

The major limitations of this study are its retrospective design and dependence solely on traditional histopathological examination using hematoxylin and eosin staining, making it difficult to exclude the true absence of lymphatic and vascular invasion in OSCC tumors. Although the criteria for 2 parameters are quite consistent, there may be significant variability in the processing of the specimens in the routine pathological practice. The thorough and serial examination of the whole cancer specimens is not usually possible in the routine pathological examination of most hospitals. These facts might affect or even underestimate the true incidence of lymphatic/vascular invasion of the OSCC tumors. Although lymphatic/vascular invasion is commonly reported on routine examination using hematoxylin and eosin staining, immunohistochemistry appears more effective for detecting lymphatic and vascular invasion.^5^ Some investigators used immunohistochemistry as a routine method of staining and reported a higher incidence of lymphatic and vascular invasion separately in OSCC using immunohistochemistry techniques (using anti-cytokeratin, CD34, a blood vessel endothelial marker, and podoplanin antibodies in 1 study, and anti-D2-40 and elastica van Gieson antibodies in the other study), but the incidence of both lymphatic and vascular invasion was also remarkably variable, as the incidence of lymphatic invasion was reported as 54% and 25%, respectively, and that of vascular invasion was reported as 29% and 49%, respectively.^36,37^ Moreover, this method is currently not commonly used by most pathologists, and there is no current consensus on the usage of any specific antibodies over than the traditional histopathological examination via hematoxylin and eosin staining for diagnosis or a standardized protocol for immunohistochemical staining. Consequently, these immunohistochemical methods are still considered investigational techniques and not included as part of the routine pathological evaluation.^38^

## CONCLUSION

Although both lymphatic and vascular invasion are associated with many clinicopathological manifestations, neither affects the risk of locoregional recurrence and distant metastasis in posttreated OSCC. Furthermore, in the survival analyses, we found that neither lymphatic nor vascular invasion is an independent prognostic indicator. Based on these results, pathological findings of either lymphatic or vascular invasion might not necessarily be an indicator for postoperative adjuvant therapy; thus, a larger prospective cohort study to investigate the utility of the sole finding of lymphatic or vascular invasion as the indication for postoperative adjuvant radiotherapy or chemoradiotherapy for patients with primary OSCC tumors is mandatory in the future.
